# Age-associated metabolic dysregulation in bone marrow-derived macrophages stimulated with lipopolysaccharide

**DOI:** 10.1038/srep22637

**Published:** 2016-03-04

**Authors:** Fan Fei, Keith M. Lee, Brian E. McCarry, Dawn M. E. Bowdish

**Affiliations:** 1 Department of Chemistry and Chemical Biology, McMaster University, Hamilton L8S4M1, Canada; 2 Department of Pathology and Molecular Medicine, Michael G. DeGroote Institute for Infectious Disease Research, McMaster University, Hamilton L8N3Z5, Canada

## Abstract

Macrophages are major contributors to age-associated inflammation. Metabolic processes such as oxidative phosphorylation, glycolysis and the urea cycle regulate inflammatory responses by macrophages. Metabolic profiles changes with age; therefore, we hypothesized that dysregulation of metabolic processes could contribute to macrophage hyporesponsiveness to LPS. We examined the intracellular metabolome of bone marrow-derived macrophages from young (6–8 wk) and old (18–22 mo) mice following lipopolysaccharide (LPS) stimulation and tolerance. We discovered known and novel metabolites that were associated with the LPS response of macrophages from young mice, which were not inducible in macrophages from old mice. Macrophages from old mice were largely non-responsive towards LPS stimulation, and we did not observe a shift from oxidative phosphorylation to glycolysis. The critical regulatory metabolites succinate, γ-aminobutyric acid, arginine, ornithine and adenosine were increased in LPS-stimulated macrophages from young mice, but not macrophages from old mice. A shift between glycolysis and oxidative phosphorylation was not observed during LPS tolerance in macrophages from either young or old mice. Metabolic bottlenecks may be one of the mechanisms that contribute to the dysregulation of LPS responses with age.

Inflammation is an evolutionarily conserved response to infection and tissue injury, which triggers a complex cascade of metabolic and genomic responses^1^. Both innate and adaptive immune function declines with age^2^,^3^,^4^, and this contributes to decreased vaccine responses^5^ and increased susceptibility to sepsis and inflammatory diseases^6^. Franceschi *et al.* proposed that macrophages play a central role in producing age-associated inflammation, which ultimately impairs the immune response^7^. Macrophages are heterogeneous tissue-resident sentinel cells that are derived from hematopoietic progenitors^8^. They initiate inflammatory responses towards microbial pathogens and repair damaged tissues^7^ by responding to their local cytokine environment and adapting to either pro-inflammatory (M1) or anti-inflammatory (M2) phenotypes^9^. With age, macrophage functions, including phagocytosis, wound healing and polarization, are impaired^10^,^11^.

Bacterial lipopolysaccharide (LPS) is a potent inflammatory stimulant that is often used to study macrophage function. Upon repeated challenge with LPS, macrophages become refractory to stimulation with LPS and this “LPS tolerance” can persist for 24–48 hrs after initial stimulation^12^. LPS tolerance is an essential immune-homeostatic response that protects against hyper-inflammatory responses during persistent infection^13^, but may alsocontribute to septic and non-infectious systemic inflammatory response syndrome (SIRS) in humans^13^. Peritoneal macrophages from young mice develop LPS tolerance more effectively than macrophages from old mice^14^. Whether failure to control inflammation due to chronic LPS exposure contributes to increased susceptibility to inflammatory diseases in old age is not known.

Inflammatory responses of macrophages can be regulated by intracellular and extracellular levels of metabolites. It is known that upon LPS stimulation, macrophages switch from oxidative phosphorylation to glycolysis as their primary energy source to sustain the increased energy demand during inflammation^15^,^16^. Enhanced glycolytic function is measured by increased levels of intra- and extra-cellular lactate. Specific transcriptional responses promoting inflammation have been shown to be regulated by metabolites such as succinate and γ-aminobutyric acid^17^. Additionally, M1/M2 polarization is regulated by increasing levels of urea cycle intermediates such as arginine, ornithine, citrulline^18^. Increased levels of adenosine as a result of inflammation can regulate inflammatory responses and are protective against tissue damage^19^. Metabolic changes have been noted in mice and humans as a result of aging^20^,^21^. Whether metabolic dysregulation can contribute to macrophage dysfunction with age is not known.

Here, for the first time, we identified age-specific metabolic dysregulation of LPS responses in bone marrow-derived macrophages. Additionally, we quantified the metabolic changes during LPS tolerance in both young and old macrophages. We discovered novel metabolites that are associated with LPS stimulation. We have found metabolic reprogramming of oxidative phosphorylation to glycolysis was suppressed in LPS stimulated macrophages from old mice. In addition, arginine metabolism, which is vital for macrophage polarization^18^,^22^, was also impaired in old macrophages. Our data indicate a possible metabolic bottleneck that prevents energy intensive inflammatory responses in old macrophages.

## Results

**Figure 1:**
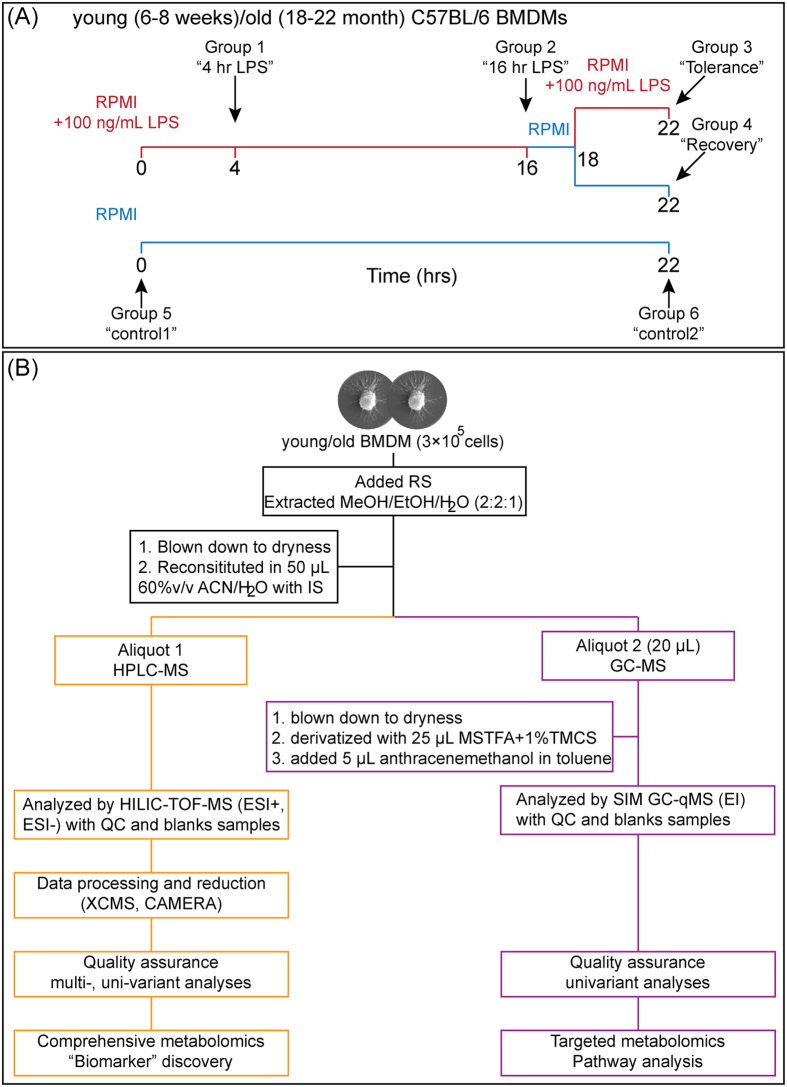
(**A**) The experimental outline. (**B**) The experimental workflow for analyzing macrophage extracts. From one macrophage culture, the sample extract was analyzed separately with HILIC-TOF-MS and GC-qMS with distinct sample preparation, data acquisition, data processing, data analysis, and quality assurance.

In order to quantitate differences in macrophage metabolism during LPS stimulation and LPS tolerance, bone marrow derived macrophages from young and old mice were analyzed using both comprehensive and targeted metabolomic strategies (Fig. 1). Liquid chromatography-mass spectrometry (LC-MS) was used to create a comprehensive metabolomic profile, which was composed of 2125 metabolite features, of which 57 polar metabolites and 64 phospholipids were identified. Gas chromatography (GC)-MS was used for targeted metabolomic analysis, which included 25 intermediates in glycolysis, the citric acid cycle (TCA), the aspartate-argininosuccinate shunt, the γ-aminobutyric acid (GABA) shunt and the urea cycle pathways (Table S1).

### Comprehensive analysis reveals novel metabolites associated with LPS responses

**Figure 2:**
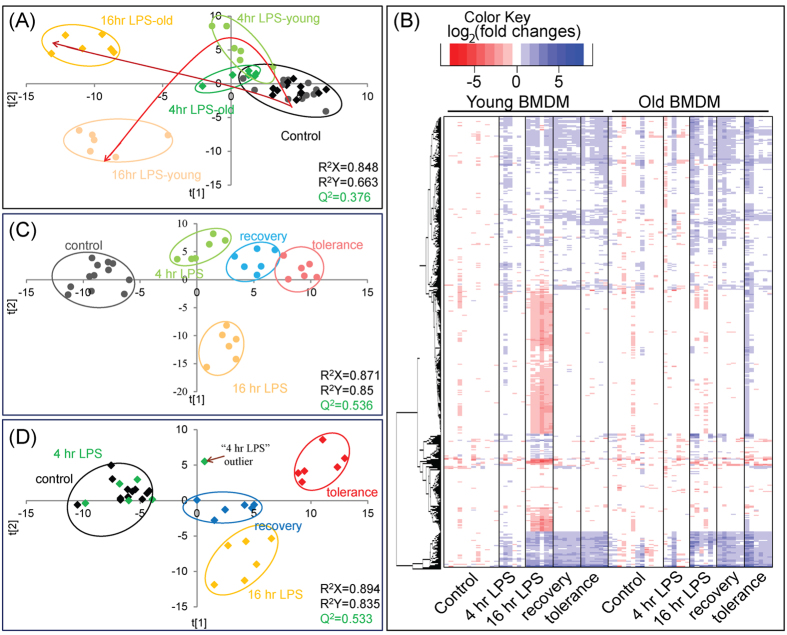
The comprehensive analyses of bone marrow-derived macrophage extracts acquired using HILIC-TOF-MS. The ionization responses of 2125 intracellular metabolite features were normalized using IS. Extracts were performed in sextuplicate with three biological replicates and two culture replicates. (**A**) OPLS-DA score plot comparing the metabolic profiles of control (0 hr), 4 hr and 16 hr LPS stimulated macrophage extracts from both young and old mice. (**B**) Heat map visualization of the intracellular metabolite changes of macrophages of young and old mice in response to LPS stimulation. The 920 significant metabolite features (p < 0.05 one-way ANOVA, fold change greater than 1.5 compared to control) are represented in rows, and the experimental conditions were listed in columns. The heat map is plotted based on log_2_(fold change) with respect to the average levels of each metabolite feature in the control of macrophages from young mice using Euclidean distance and complete-linkage clustering. OPLS-DA score plot comparing the metabolic profiles of control (0 hr), 4 hr, and 16 hr LPS stimulated macrophage extracts as well as “recovery” and “tolerance” treated macrophage extracts from (**C**) young or (**D**) old mice. Controls 1 and 2 obtained at the beginning (t = 0 hr) and the end of the experiments (t = 22 hrs) were considered as a single group. Samples belonging to the same treatment group were highlighted by open circles.

**Figure 3:**
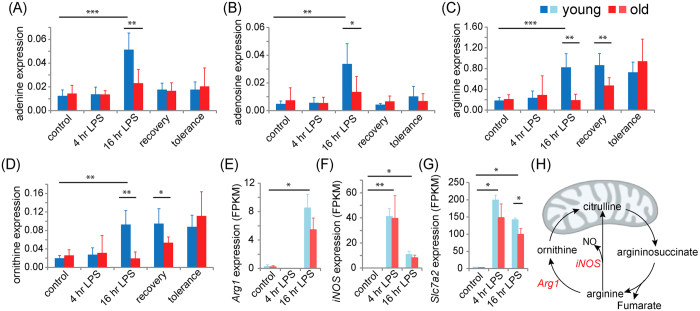
The relative intracellular levels and gene expression of selected metabolites and genes. The intracellular levels of adenine (**A**), adenosine (**B**), arginine (**C**) and ornithine (**D**) were acquired at control (0 hr), 4 hr and 16 hr of LPS stimulation as well as at the “recovery” and “tolerance” conditions for macrophages of both young and old mice. The gene expressions of *Arg1* (**E**), *iNOS* (**F**) and *Slc7a2* (**G**) were acquired at control (0 hr), 4 hr and 16 hr of LPS stimulation for macrophages of young and old mice. The urea pathway was illustrated in (**H**). Metabolomics and gene expression data of macrophages from young mice were labelled in shades of blue; those from old mice were labelled in shades of red. *p < 0.05; **p < 0.01; ***p < 0.001.

The metabolome of bone marrow derived macrophages from young mice were analyzed and compared at 0, 4 and 16 hr of LPS stimulation. To ensure any metabolic changes only resulted from LPS stimulation and were not a result of the 22 hr incubation, we compared the metabolic profiles of unstimulated macrophages at t = 0 hr and t = 22 hrs. Less than 1.3% of the metabolic features showed any significant change over the 22 hr period. Significant metabolic changes were observed for LPS stimulated macrophages after 4 hrs of stimulation, and a more dramatic change was noted after 16 hrs of LPS stimulation (Fig. 2A). The metabolite features from young macrophages that were differentially expressed after 4 hrs of LPS stimulation were compared to the unstimulated control 4.5% (96/2125) (Fig. 2B). After 16 hrs of LPS stimulation, over half of the metabolic features (1081/2125) were significantly altered in the young macrophages. Of the differentially expressed features, 27.2% (579/2125) showed increased expression and 23.6% (502/2125) features were reduced compared to the unstimulated control. Metabolites that were found to increase in macrophages of young mice after 16 hrs of LPS stimulation included adenine, adenosine, ornithine, arginine (Fig. 3A–D), pantothenic acid, uridine diphosphate N-acetylglucosamine (UDP-GlcNAc), N-acetyl-phenylalanine, taurine, hypotaurine, UDP-glucose (UDP-G), glucosamine-6-phosphate (GlcN6P), methyl-malonic acid, lysine, proline, glutamine, phosphatidylglycerols (PGs), phosphatidylethanolamine (PEs) and phosphatidylglycerols (PCs). N-acetyl glutamic acid and N-acetyl-aspartic acid were reduced after 16 hrs of LPS exposure. Most metabolic features remained unidentified. The comprehensive metabolomic approach allows the discovery of novel metabolites associated to macrophage LPS responses (normalized levels of metabolites are included in the supplementary material 2).

### Macrophage metabolism in response to LPS decreases with age

The metabolomes of bone marrow derived macrophages from both young and old mice were analyzed and compared prior to LPS stimulation (t = 0 hr), and after 4 and 16 hrs of LPS stimulation. Only 0.4% of the metabolites were significantly different between macrophages derived from young and old mice in the unstimulated controls indicating that there were virtually no detectable age-associated metabolic differences in the steady state. Age-associated differences in metabolism after LPS stimulation were visualized using an OPLS-DA score plot (Fig. 2A). Old macrophages were essentially non-responsive to LPS stimulation as compared to the young. After 4 hrs of LPS stimulation metabolic changes were apparent between young and old macrophages, and these became more distinct after 16 hrs of LPS stimulation. Unlike LPS stimulated young macrophages where approximately half of the metabolome was altered, only 2.2% (46/2125) and 13.3% (282/2125) of the features were altered in the old macrophages after 4 hrs and 16 hrs of LPS stimulation, respectively. There were however, 10.0% (211/2125) of the metabolic features were significantly changed with similar magnitude in both young and old macrophages after 16 hrs of LPS stimulation. Metabolites that were found to be increased in both young and old macrophages after 16 hrs of LPS stimulation included pantothenic acid, UDP-GlcNAc, N-acetyl-phenylalanine, PEs and almost half of detectable PCs. Overall, metabolic responses to LPS stimulation decrease with age.

### Macrophages from old mice have defects in core metabolism during LPS stimulation

Macrophages switch their core metabolism from oxidative phosphorylation to glycolysis when stimulated with LPS^15^,^16^. To examine whether the core metabolism of activated macrophages was affected by age, we designed a targeted metabolomics approach including in intermediates in glycolysis, the TCA cycle, the GABA shunt, the aspartate-argininosuccinate shunt and the urea cycle.

**Figure 4:**
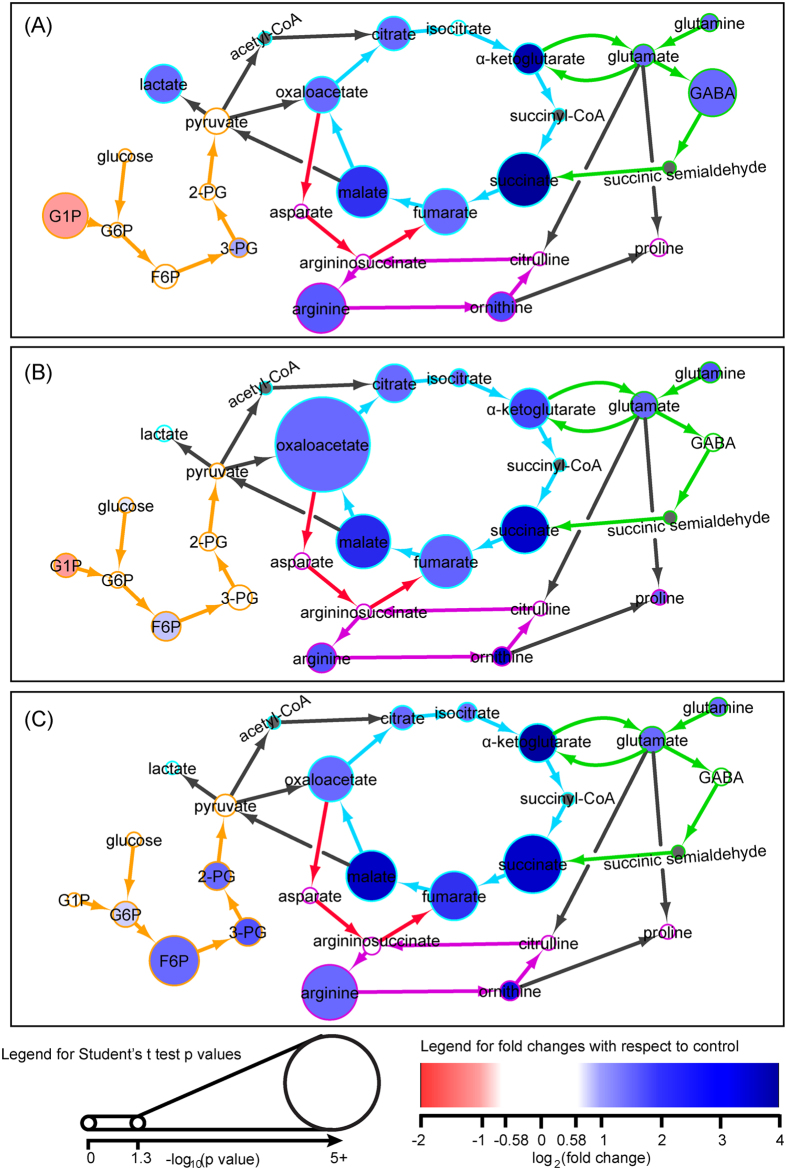
The enrichment map of glycolysis, the TCA cycle, the GABA shunt, and the urea cycle intermediates in bone marrow-derived macrophages from young mice in response to (**A**) 16 hr LPS stimulation, (**B**) “recovery”, and (**C**) “tolerance” as compared to unstimulated macrophages. The network of metabolite interactions was built based on the BioCyc database and pathway published from Jha *et al.*^25^. The node size is proportional to the significance of metabolite changes compared to the control. The colors of the nodes indicate the log_2_(fold changes) of metabolite levels of each experimental condition compared to the control with a decrease colored in red and an increase colored in blue. Acetyl-CoA, succinyl-CoA and succinic semialdehyde are not detected and therefore are labelled in grey. The glycolysis pathway is labelled in orange; the TCA cycle is labelled in blue; the GABA shunt is labelled in green; the glutamate-argininosuccinate shunt is labelled in red; the urea cycle is labelled in purple.

**Figure 5:**
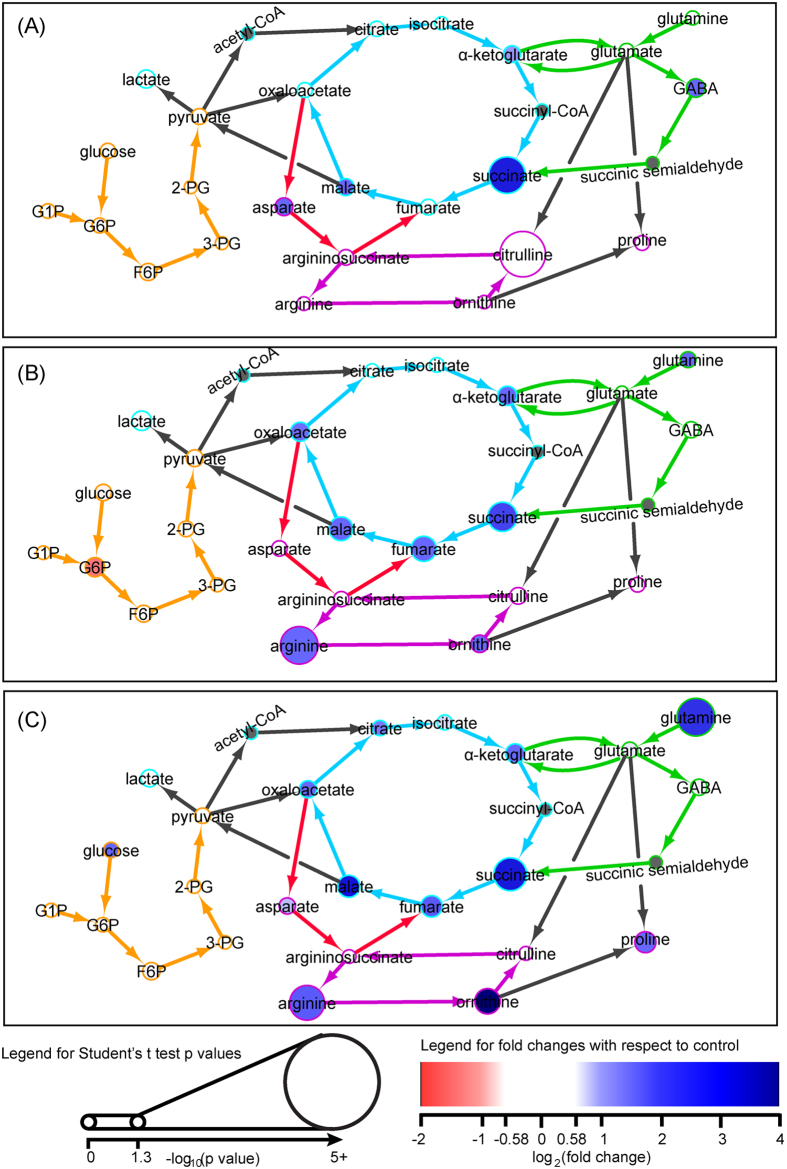
The enrichment map of glycolysis, the TCA cycle, the GABA shunt, and the urea cycle intermediates in bone marrow-derived macrophages from old mice in response to (**A**) 16 hr LPS stimulation, (**B**) “recovery”, and (**C**) “tolerance” as compared to unstimulated macrophages. The network of metabolite interactions was built based on the BioCyc database and pathway published from Jha *et al.*^25^. The node size is proportional to the significance of metabolite changes compared to the control. The colors of the nodes indicate the log_2_(fold changes) of metabolite levels of each experimental condition compared to the control with a decrease colored in red and an increase colored in blue. Acetyl-CoA, succinyl-CoA and succinic semialdehyde are not detected and therefore are labelled in grey. The glycolysis pathway is labelled in orange; the TCA cycle is labelled in blue; the GABA shunt is labelled in green; the glutamate-argininosuccinate shunt is labelled in red; the urea cycle is labelled in purple.

After 4 hrs of LPS stimulation, metabolites associated with the TCA cycle including oxaloacetate, malate, fumarate, succinate, α-ketoglutarate, citrate and glutamate were increased in macrophages from young mice (Figs S2A and S3A). After 16 hrs of LPS stimulation, the above-named metabolites remained elevated, and in addition, fructose-6-phosphate, lactate, GABA, arginine and ornithine also increased compared to the unstimulated control (Fig. 4A). In contrast, very few changes in this core metabolic pathway were noted in macrophages from old mice after LPS stimulation. Isocitrate, 2-phosphoglycerate (2PG) and 3PG were only decreased in the old macrophages after 4 hrs of LPS stimulation, and after 16 hrs of LPS stimulation, only arginine, malate, aspartate and GABA were increased (Fig. 5A, S2B, S3B). Decreased glucose-1-phosphate was observed in macrophages from both young and old mice after 4 and 16 hrs of LPS stimulation.

### Metabolic changes during LPS tolerance

To examine the metabolic response associated with LPS tolerance in macrophages, we analyzed the metabolome of macrophages stimulated with a second dose of LPS for 4 hrs (“tolerance”). As a control, after 16 hrs of LPS stimulation, the cells were washed and cultured in LPS-free medium for 6 hrs (“recovery”). As visualized by the OPLS-DA score plot, the “recovery” and “tolerance” metabolic profiles from young mice resembled the early stage of LPS stimulation (Fig. 1C). In contrast, these profiles from old macrophages were distinct from 4 and 16 hrs of LPS stimulation (Fig. 1D).

We confirmed the LPS tolerance by quantifying transcripts that were known to be associated with the LPS tolerance (Fig. S4). Some metabolic changes appeared to be reversible when LPS was removed, whereas others were irreversible. Reversible metabolites were induced upon the first LPS stimulation but whose levels returned to baseline when LPS were removed. Reversible metabolites did not change expression levels after and LPS re-stimulation, however. Irreversible metabolites were induced upon primary LPS stimulation and remained elevated during the LPS re-stimulation. There are also a unique set of metabolites that whose exposure only changed during LPS re-stimulation (i.e. in the tolerant macrophages). For macrophages from young mice, 20.0% (424/2125) of the metabolic features were irreversible, 30.9% (657/2125) were reversible, and 9.4% (200/2125) of the features were differently expressed after LPS re-stimulation (i.e. in tolerant macrophages). Metabolites that were increased in primary LPS stimulation and remained elevated in both the “recovery” and “tolerance” group included taurine, hypotaurine, UDP-G, GlcN6P, lysine, proline, ornithine, arginine, glutamine, PEs and PCs. Changes in PG levels also increasedduring LPS tolerance, although the magnitude of induction during LPS re-stimulation was much lower compared to after the 16 hr of LPS stimulation. Conversely, for macrophages from old mice, 6% (128/2125) of the metabolic features were irreversible, 7.2% (154/2125) were reversible, and 6.8% (145/2125) of the features were only significantly different in the tolerance group. Interestingly, arginine, ornithine, UDP-GlcNAc, UDP-G, ornithine, glutamine and hypotaurine, which were only elevated in the young macrophages after 4 and 16 hrs of LPS stimulation, were found elevated in the “recovery” and “tolerance” groups of the old macrophages. This suggests that the metabolic responses to LPS stimulation are delayed in old macrophages.

Similar trends were also observed for metabolites associated with macrophage core metabolism. In young macrophages, almost all of the metabolites that were elevated after 16 hrs of LPS stimulation were still elevated in the “recovery” and “tolerance” groups. These included citrate, oxaloacetate, malate, fumarate, succinate, α-ketoglutarate, arginine, ornithine, glutamate and glutamine. Only GABA and lactate decreased to the levels observed in unstimulated macrophages. Interestingly, proline levels did not increase during LPS stimulation, but increased in the “recovery” group. Intermediates of the glycolysis pathway including isocitrate and glycolysis intermediates such as glucose-6-phosphate (G6P), fructose-6-phosphate (F6P), 3PG and 2PG were induced only after secondary LPS exposure in the young macrophages. Metabolic responses to LPS were delayed in the old macrophages. Metabolites such as oxaloacetate, fumarate, glutamine, arginine and ornithine were only increased in old macrophages after 16 hrs of LPS stimulation and in both the “recovery” and “tolerance” macrophages.

### Age-associated changes in arginine metabolism

Macrophage polarization requires arginine metabolism via the urea cycle^18^. The urea cycle intermediates, arginine and ornithine, were increased in the young macrophages after 16 hrs of LPS stimulation (Fig. 4). The levels of arginine and ornithine remained elevated in the young macrophages when LPS was removed and also during LPS tolerance. Similar to changes observed in macrophage core metabolism, there was a delay in arginine metabolism in old macrophages. Increasing levels of arginine and ornithine were only observed during the “recovery” and “tolerance” samples (Fig. 5).

The gene expression of *Arg1*, *iNOS* (*Nos2*) and the cationic amino acid transport (*Slc7a2*) were quantified. The expression of *Arg1* and *iNOS* were elevated but not statistically different between LPS stimulated macrophages from young and old mice. Only *Slc7a2* was differentially expressed between young and old macrophages after 16 hrs of LPS exposure, for which the expression of *Slc7a2* in young macrophages was 1.43-fold greater than the old (Fig. 3E–H).

## Discussion

LPS stimulation triggers a shift in macrophage core metabolism from oxidative phosphorylation to glycolysis^15^,^16^. Glycolysis occurs in the cytoplasm and produces two ATPs per glucose. The end product of glycolysis, pyruvate, enters the mitochondria and initiates the TCA cycle and oxidative phosphorylation and results in the production of an additional 36 ATPs under aerobic conditions. Under anaerobic conditions, pyruvate is reduced to lactate in the cytoplasm and secreted^23^. Although only 5% of the glucose’s energy potential is taken advantage of during glycolysis, it can produce ATP at a much faster rate than oxidative phosphorylation. In addition to glucose metabolism, glutamine contributes to a third of the energy requirement of unstimulated macrophages via glutamine anaplerosis^24^. The metabolic switch to glycolysis during LPS stimulation is a rapid way to accommodate the increased energy demand during inflammation. As macrophages shift from using oxidative phosphorylation to glycolysis, lactate and TCA intermediates such as malate, citrate and fumarate are increased intracellularly^15^,^16^. Consistent with this, we have observed increasing levels of those metabolites in young macrophages after 16 hrs of LPS stimulation.

Macrophage specific adaptation of the TCA cycle has beenreported. Jha *et al.* have reported that in macrophages, the metabolic flow between isocitrate and α-ketoglutarate is disrupted due to aM1 macrophage specific metabolic break-point in the TCA cycle^25^. Consistent with this result, in young macrophages, we also observed a metabolic break in the TCA cycle between citrate and α-ketoglutarate where, despite a global increase of almost all the TCA cycle intermediates, isocitrate remained unchanged after 16 hrs of LPS stimulation. Since we also observed this in our model of inflammation, this break-point in the TCA cycle may not be specific to M1 phenotypes but rather an indication of energy metabolism during inflammation. We did not observe the citrate/α-ketoglutarate metabolic break in old macrophages because the TCA cycle remained effectively unchanged after LPS stimulation.

In the macrophages from old mice, the core metabolism was mostly unchanged in response to LPS stimulation, suggesting that mitochondrial function may be impaired. Mitochondrial dysfunction during aging is well documented^26^. Although mitochondrial metabolic dysfunction has not been thoroughly studied in macrophages, several studies of mitochondria from muscle tissues have shown reduced rates of glycolysis and the TCA cycle with age^27^,^28^. We did not detect increasing levels of lactate, malate, fumarate and citrate in LPS stimulated macrophages from old mice, consistent with mitochondrial dysfunction and an inability to shift from oxidative phosphorylation to glycolysis.

Interestingly, succinate was the only TCA cycle intermediate that was elevated in macrophages from both young and old mice in response to LPS stimulation (3.58- and 2.73- fold change at 16 hrs LPS respectively). Elevated succinate in response to LPS stimulation has been shown to stabilize hypoxia-inducible factor-1α (HIF-1α), a transcription factor that is required for IL-1β production^17^. IL-1β is a key inflammatory cytokine produced by macrophages during inflammatory responses. The induction of glutamine anaplerosis and the GABA shunt pathways are the principle source of succinate^17^. In young macrophages, we have observed consistently elevated levels of GABA (2.37-fold), glutamate (2.38-fold) and glutamine (2.66-fold) that fed into succinate production. In contrast, only GABA (2.70-fold) was increased in old macrophages after LPS stimulation. Although there are conflicting reports as to whether old macrophages produce more or less inflammatory cytokines in response to LPS^11^,^29^, our data imply succinate and its biosynthetic pathway would not be a rate limiting factor in generating an inflammatory response.

Arginine is required for macrophage activation^22^. Elevated levels of arginine and ornithine were observed in young but not old macrophages following 16 hrs of LPS stimulation. Intracellular arginine is mostly imported from the extracellular environment via cationic amino acid transport (CAT) in both humans and mice^30^,^31^. Of all the genes encoding the CAT, *Slc7a2* is the only gene whose expression is inducible and is required during both M1 and M2 macrophage polarization^31^,^32^. We have observed a 30% reduction in *Slc7a2* expression in the old macrophages compared to the young after 16 hrs of LPS stimulation, which likely contributes to the lower intracellular arginine level in the old macrophages. Arginine may also be synthesized via the aspartate-argininosuccinate shunt, which joins the TCA cycle with the urea cycle^25^. Inhibition of the aspartate-argininosuccinate shunt inhibits M1 polarization with low *iNOS* expression and nitric oxide production^25^. However, in our study, 16 hrs of LPS stimulation did not affect the aspartate-argininosuccinate shunt (i.e. aspartate, argininosuccinate) in macrophages from both young and old mice. Therefore, the aspartate-argininosuccinate shunt is unlikely to contribute to the increase of intracellular arginine in activated macrophages.

Metabolism of arginine via *Arg1* or *iNOS* in the urea cycle regulates macrophage polarization^18^ M1 macrophages express *iNOS* which metabolizes arginine to nitric oxide to prevent pathogen infection by limiting free arginine and producing nitric oxide, a powerful antimicrobial agent. M2 macrophages express *Arg1* which hydrolyzes arginine to ornithine to stimulate cell division and tissue repair through the production of polyamines and proline. LPS is known to induce both *Arg1* and *iNOS* 9, which we have also observed in LPS stimulated macrophages from young and old mice. Gene expression of *Arg1* and *iNOS* were measured to further characterize the role of the urea cycle in age (Fig. 3H). Consistent with our data, there were no age-associated changes in *iNOS* or *Arg1* expression in bone marrow-derived macrophages; however, others have reported decreased expression in LPS stimulated splenic and peritoneal murine macrophages^11^. Therefore, arginine metabolism is unlikely to be the rate limiting factor in the LPS response of old macrophages.

Dysregulation of LPS tolerance has been proposed as a contributing factor to the increased susceptibility of the elderly to sepsis and inflammatory disorders^13^. To test whether macrophages from old mice have defects in LPS tolerance, we measured a well-defined set of pro-inflammatory and anti-microbial genes associated with this phenomenon^12^. Both the young and old macrophages experienced LPS tolerance equally at the transcriptional level. However, our data demonstrated that there were metabolic changes specific to LPS tolerance. In the young macrophages, lactate levels were increased after the initial LPS stimulation, but returned to baseline levels when LPS was removed and did not increase during the second LPS stimulation. This indicated that the core metabolism of young macrophages did not shift from oxidative phosphorylation to glycolysis during LPS tolerance. This inability to adjust macrophage core metabolism to during LPS tolerance may be compensated for by the up-regulation of glycolysis as indicated by the elevated levels of glycolysis intermediates. The inability to switch from glycolysis to oxidative phosphorylation may be a key metabolic break in LPS tolerance. In contrast to young macrophages, old macrophages did not shift from oxidative phosphorylation to glycolysis in either the first or second LPS stimulation. Moreover, elevated levels of arginine and TCA cycle intermediates were detected in both the first and second LPS stimulation in the young macrophages. In old macrophages, those metabolites were only induced much later, indicating that the old macrophages had a delayed metabolic response to LPS stimulation. Whether this delay is because of age-associated deterioration in mitochondria function, and specifically ATP production from glucose, is worth future investigation.

Adenosine is a “retaliatory metabolite” whose intracellular level is amplified at sites of injury and inflammation, and also mediates the resolution of inflammation by limiting tissue destruction^19^. Utilization of ATP during macrophage activation as a result of high metabolic activity leads to increased levels of intracellular adenosine^33^. Moreover, elevated levels of adenosine are known to enhance glycolysis and ATP production that supports metabolism in activated macrophages^33^. When adenosine is expressed in excess, it binds to adenosine receptors to suppress inflammatory responses, which preserves tissue homeostasis and prevents tissue damage^19^. Preventing adenosine breakdown by inhibiting adenosine deaminase reduces systemic inflammation such as sepsis^34^. We observed increasing intracellular levels of adenosine and its precursor, adenine, after LPS stimulation in young macrophages. Young macrophages reduced the expression of adenosine and adenine to the baseline level of unstimulated macrophages during LPS tolerance. In contrast, levels of adenosine and adenine were not changed during LPS stimulation or tolerancein old macrophages. This might be a result of the decreased metabolic activity and reduced rate of glycolysis in old macrophages.

Overall, age-associated metabolic dysfunction was observed in bone marrow-derived macrophages after LPS stimulation and during LPS tolerance. Inflammatory responses are energetically costly and result in high metabolic activity^35^. For example, a 1 °C to 4 °C rise in core body temperature during fever helps to resolve bacterial and viral infections, and a 1 °C increase in temperature demands a 10–12.5% increase in metabolic rate^36^. However, fever responses are often absent or blunted in the elderly^37^, and rapid muscle wasting to sustain the high energy demand is common^38^. We have observed metabolic bottlenecks between the switch from oxidative phosphorylation to glycolysis, which might contribute to impaired LPS-induced inflammation in the elderly. Moreover, the elderly are more vulnerable to nutrient deficiencies, such as arginine^39^. Low arginine can attribute to the retarded immune function^22^ and is associated to poor health outcomes in the elderly^40^. We found old macrophages are unable to adjust intracellular arginine level in response to LPS stimulation, but whether this is associated with arginine-related immune deficiencies is not known. In this study, metabolic bottlenecks were observed for macrophages from old mice during LPS stimulated inflammatory events, and metabolic dysregulation should be considered as a possible mechanism for declining immune function with age.

## Experimental procedures

### Bone marrow-derived macrophage culture

Bone marrow progenitors were collected from the femurs and tibia of young (6–8 wk) and old (18–22 mo) C57BL/6 mice (The Jackson Laboratory, Maine, USA). Progenitor cells were cultured for seven days in RPMI-1640 supplemented with 10% fetal bovine serum (FBS), 1% L-glutamine, 1% penicillin/streptomycin and 15% L929 fibroblast cell conditioned medium on 150 mm Petri dish (Fisherbrand) as per standard protocols^41^,^42^. On day 8, bone marrow-derived macrophages were counted and seeded into 24-well tissue-culture-treated plates (Falcon) and incubated overnight. For metabolomic studies, three biological replicates were performed. For each biological replicate, 3 × 10^5^ macrophages were seeded per well in 1 mL RPMI-1640 with two culture replicates per mouse (n = 6 per treatment). For gene expression study, three biological replicates were performed. For each biological replicate, 1 × 10^6^ macrophages were seeded per well in 3 mL RPMI-1640 (n = 3 per treatment). All animal studies were approved by and performed in accordance with McMaster’s Animal Research Ethics Board.

### Macrophage LPS stimulation

Macrophages from both young and old mice were divided into six treatment groups. Groups 1 and 2 were stimulated with 100 ng/mL of bacterial lipopolysaccharide (LPS, Ultrapure LPS, *E. coli* 0111:B4 from Invivogen) in RPMI-1640 for 4 and 16 hrs respectively. Group 3 (“tolerance”) was incubated for 16 hrs with an initial LPS challenge (100 ng/mL), washed in PBS, incubated in regular RPMI-1640 for 2 hrs, and then re-stimulated with 100 ng/mL of LPS for 4 hrs. Group 4 (“recovery”) was incubated for 16 hrs with LPS (100 ng/mL), washed in PBS, incubated in regular RPMI-1640 for 6 hrs. Group 5 and 6 were controls that were cultured in regular RPMI-1640 for 0 and 22 hrs. The experimental protocol for LPS tolerance study was based on Foster *et al.*^12^. All cells were washed once with cold phosphate-buffered saline (PBS) and collected for metabolomic and gene expression studies.

### Macrophage extraction for metabolomic analyses

After macrophages were washed with 1 mL of cold PBS, the cells were detached from the 24-well plate using a cell lifter in the presence of cold extraction solvent mixture methanol/ethanol/H_2_O (200 μL, 2:2:1, v/v) containing standards for recovery determination (98% L-methionine-d_3_, 98% L-tryptophan-d_5_)^43^. The cell suspension was transferred into a 1.5 mL microtube (Diamed, ON, Canada) and vortex mixed for 2 min in the presence of two 2 mm ball bearings. After removal of the bearings, the mixture was centrifuged at 9500 × g for 3 min at 4 °C. The supernatant was collected and the precipitated pellet (containing DNA, RNA, and proteins) was re-extracted twice with cold methanol/ethanol/H_2_O (50 μL) as with above. Cell extract (150 μL) was dried under nitrogen gas and re-solubilized in 60%v/v ACN/H_2_O containing standards (50 μL, 98% L-phenylalanine-d_8_, diphenylalanine, glycine-phenylalanine) for peak area normalization (IS) for LC-MS analysis. A quality control pooled sample was prepared by combining ACN/H_2_O macrophage extracts (5 μL) from a total of 72 samples of all treatment groups.

For GC-MS analysis, 20 μL of the ACN/H_2_O macrophage extract or pooled samples was dried under nitrogen gas, and reconstituted in 25 μL of 1%v/v chlorotrimethylsilane (TMCS) in N-methyl-N-(trimethylsily)trifluoroacetamide (MSTFA) and 5 μL anthracenemethanol (IS for GC-MS) in toluene (1.2 ng/μL). The samples were incubated at 60 °C for 1 hr and analyzed by GC-MS immediately.

The entire sample preparation procedure was performed on ice or in a cold room. The sample extracts were stored in a −80 °C freezer prior to analyses.

### LC-HILIC-TOF-MS comprehensive analysis

Macrophage extracts were analyzed using a Agilent Technologies Model 1200RR series II liquid chromatograph coupled to a Bruker micrOTOF II Mass Analyzer as previously described^43^. A Phenomenex Kinetix 2.6 μm core shell HILIC column (2.1 × 50 mm, pore size 100 Å) was operated at 200 μL/min using a linear gradient of acetonitrile (A) and 10 mM ammonium acetate, adjusted to pH 3 (B). The column temperature was maintained at 40 °C, and the auto sampler storage tray was set at 4 °C. LC gradient: 0–0.5 min, 95% A; 0.5–12.5 min, 95% A to 35% A; hold at 35% A for 0.5 min; 35% A to 95% A over 1 min; re-equilibration at 95% A for 10 min prior to the next injection. A 2 μL sample was injected to a total run time of 24 min for both positive and negative electrospray ionization (ESI) modes. The mass spectrometer setting was identical to those previously reported in Fei *et al.*^43^.

### GC-MS targeted analysis

GC-MS analyses were performed using an Agilent 6890 N gas chromatograph (Santa Clara, CA, USA), equipped with a DB-17ht column (30 m × 0.25 mm i.d. ×0.15 μm film, J & W Scientific) and a retention gap (deactivated fused silica, 5 m × 0.53 mm i.d.), and coupled to an Agilent 5973 MSD single quadruple mass spectrometer. The autosampler storage tray was maintained below 5 °C with a cooler system. The derivatized macrophage extract (1 μL) was injected using Agilent 7683 autosampler in splitless mode. The injector temperature was 230 °C and carrier gas (helium) flow was 0.8 mL/min. The transfer line was 280 °C and the MS source temperature was 230 °C. The column temperature was set at 70 °C for 0.1 min, raised to 225 °C at 5 °C/min, and then 310 °C at 55 °C/min and held there for 4 min. After a five minute solvent delay, mass spectra were acquired using electron ionization (EI) with a selected-ion-monitoring (SIM) mode as in Table S2. Metabolic intermediates of glycolysis, TCA, aspartate-argininosuccinate shunt, GABA shunt and urea cycle were included in this study (Table S1).

### Quality Control

For both LC-MS and GC-MS metabolomic analyses, pooled samples were run 7 times to condition the column prior to sample analysis and also run after every 5^th^ sample. MeOH/EtOH/H_2_O blank and a standard mixture containing IS and RS were also run after every 10 samples. All samples were run in a randomized sequence.

### Gene expression analysis

Modified from previously published protocol^44^, the total RNA of macrophages was extracted and purified using TRIzol (Invitrogen, Carlsbad, CA, USA) and RNeasy Mini Kit (Qiagen, Venlo, Netherlands). Ribosomal RNA was depleted using the Human/mouse/Rat RiboZero Magnetic Kit (Epicentre, Madison, WI, USA), and verified using the Agilent RNA 6000 Nano Kit. DNases were removed using Turbo DNase (Invitrogen), and the sample was purified using RNAClean XP beads. The first strand of cDNA was synthesized using Superscript III (Invitrogen). Complimentary second strand cDNA was synthesized with RNase H and Klenow fragment of DNA polymerase I (Invitrogen). The cDNA was sonicated into 150 base pair fragments using a Covaris S220 Focused-ultrasonicator and deoxyadenosine monophosphate was incorporated to the cDNA fragment using NEBNext dA-Tailing Module (New England Biolabs). The cDNA library was sequenced using the Illumina HiSeq system.

### Data Analyses and metabolite identification

The comprehensive LC-MS data were processed as in Fei *et al.*^43^. The LC-MS spectra were converted to. mzXML format using Bruker CompassXport followed by intermal mass calibration using sodium formate cluster in both ESI+ and ESI− modes by Bruker’s DataAnalysis 4.0 SP4. The metabolite features were extracted and aligned using open source XCMS with centWave algorithm (minfrac = 0.8)^45^; adducts, isotopic ions, and in-source fragments were identified using CAMERA^46^. The metabolite features were normalized with IS eluted closest to their retention time (i.e. features eluted before 6.50 min were normalized by phe-phe; features eluted between 6.50 and 7.80 min were normalized by L-phenylalanine-d_8_; features eluted after 7.80 min were normalized by gly-phe). After data reduction, the final data set was composed of 2125 metabolic features (Table S4).

The metabolite features were identified by matching the mass-to-charge (m/z) and the retention time of authentic standards or compound analogs (for phospholipid identification only). There were 150 features identified to 121 metabolites in the final data set, 57 were polar metabolites and 64 were phospholipids. One metabolite could have multiple metabolic features resulting from adducts or in-source fragments.

For the GC-MS dataset, peak detection and spectrum deconvolution were processed using Agilent’s Enhanced ChemStation. Multiple peaks generated from direct derivitization of a single metabolite were combined. The peak area of metabolites was normalized to anthracenemethanol. The final data sets for comprehensive LC-MS and targeted GC-MS analyses are included in supplementary material 2.

### Statistical Analyses

The final data set of comprehensive LC-MS analysis was analyzed using multivariate analysis including principal component analysis (PCA) and OPLS-DA after pareto scaling by SIMCA-P + 12.0.1 (Umetrics, Umeå, Sweden). The normality of both LC-MS and GC-MS data were analyzed using Kolmogorov-Smirnov test by SPSS 20 (SPSS, Chicago, IL, USA). Both LC-MS and GC-MS data were analyzed with univariate statistical tool including Student’s t test (two-tailed, unpaired heteroscedastic) and one-way ANOVA by Microsoft Excel 2010 and MetaboAnalyst 3.0, respectively. Metabolic features or metabolites with p value less than 0.05 and fold change greater than 1.5 between treatment groups were considered significantly differentiated. Heatmap was generated with R Project 2.12.2 using gplots.

## Additional Information

**How to cite this article**: Fei, F. *et al.* Age-associated metabolic dysregulation in bone marrow-derived macrophages stimulated with lipopolysaccharide. *Sci. Rep.* **6**, 22637; doi: 10.1038/srep22637 (2016).

## Supplementary Material

Supplementary Information

## References

[b1] Nathan C. Points of control in inflammation. Nature 420, 846–852 (2002). 12490957 10.1038/nature01320

[b2] Solana R., Pawelec G. & Tarazona R. Aging and innate immunity. Immunity 24, 491–494 (2006). 16713963 10.1016/j.immuni.2006.05.003

[b3] Weng N.-P. Aging of the immune system: how much can the adaptive immune system adapt? Immunity 24, 495–499 (2006). 16713964 10.1016/j.immuni.2006.05.001 PMC2266981

[b4] Gomez C. R., Boehmer E. D. & Kovacs E. J. The aging innate immune system. Curr. Opin. Immunol. 17, 457–462 (2005). 16084711 10.1016/j.coi.2005.07.013

[b5] Weiskopf D., Weinberger B. & Grubeck-Loebenstein B. The aging of the immune system. Transpl. Int. 22, 1041–1050 (2009). 19624493 10.1111/j.1432-2277.2009.00927.x

[b6] Ginaldi L., Loreto M. F., Corsi M. P., Modesti M. & De Martinis M. Immunosenescence and infectious diseases. Microbes. Infect. 3, 851–857 (2001). 11580980 10.1016/s1286-4579(01)01443-5

[b7] Franceschi C. *et al.* Inflamm-aging: An evolutionary perspective on immunosenescence. Ann. N. Y. Acad. Sci. 908, 244–254 (2000). 10911963 10.1111/j.1749-6632.2000.tb06651.x

[b8] Gordon S. & Taylor P. R. Monocyte and macrophage heterogeneity. Nat. Rev. Immunol. 5, 953–964 (2005). 16322748 10.1038/nri1733

[b9] Murray P. J. *et al.* Macrophage activation and polarization: nomenclature and experimental guidelines. Immunity 41, 14–20 (2014). 25035950 10.1016/j.immuni.2014.06.008 PMC4123412

[b10] Plowden J., Renshaw-Hoelscher M., Engleman C., Katz J. & Sambhara S. Innate immunity in aging: impact on macrophage function. Aging cell 3, 161–167 (2004). 15268749 10.1111/j.1474-9728.2004.00102.x

[b11] Mahbub S., Deburghgraeve C. R. & Kovacs E. J. Advanced Age Impairs Macrophage Polarization. J. Interferon Cytokine Res. 32, 18–26 (2012). 22175541 10.1089/jir.2011.0058 PMC3255514

[b12] Foster S. L., Hargreaves D. C. & Medzhitov R. Gene-specific control of inflammation by TLR-induced chromatin modifications. Nature 447, 972–978 (2007). 17538624 10.1038/nature05836

[b13] Cavaillon J.-M. & Adib-Conquy M. Bench-to-bedside review: Endotoxin tolerance as a model of leukocyte reprogramming in sepsis. Crit. Care 10, 233 (2006). 17044947 10.1186/cc5055 PMC1751079

[b14] Sun Y. *et al.* Effects of aging on endotoxin tolerance induced by lipopolysaccharides derived from Porphyromonas gingivalis and Escherichia coli. PLoS one 7, e39224 (2012). 22723968 10.1371/journal.pone.0039224 PMC3377652

[b15] Rodríguez-Prados J.-C. *et al.* Substrate fate in activated macrophages: a comparison between innate, classic, and alternative activation. J. Immunol. 185, 605–614 (2010). 20498354 10.4049/jimmunol.0901698

[b16] Sugimoto M. *et al.* Non-targeted metabolite profiling in activated macrophage secretion. Metabolomics 8, 624–633 (2011).

[b17] Tannahill G. M. *et al.* Succinate is an inflammatory signal that induces IL-1β through HIF-1α. Nature 496, 238–242 (2013). 23535595 10.1038/nature11986 PMC4031686

[b18] Mills C. D., Kincaid K., Alt J. M., Heilman M. J. & Hill A. M. M-1/M-2 Macrophages and the Th1/Th2 Paradigm 1. J. Immunol. 164, 6166–6173 (2000).10843666

[b19] Haskó G. & Cronstein B. N. Adenosine: an endogenous regulator of innate immunity. Trends Immunol. 25, 33–39 (2004). 14698282 10.1016/j.it.2003.11.003

[b20] Houtkooper R. H. *et al.* The metabolic footprint of aging in mice. Sci. Rep. 1, 134 (2011). 22355651 10.1038/srep00134 PMC3216615

[b21] Yu Z. *et al.* Human serum metabolic profiles are age dependent. Aging cell 11, 960–967 (2012). 22834969 10.1111/j.1474-9726.2012.00865.x PMC3533791

[b22] Hibbs J. B. Jr, Vavrin Z. & Taintor R. R. L-Arginine is required for expression of the activated macrophage effector mechanism causing selective metabolic inhibition in target cells. J. Immunol. 138, 550–565 (1987). 2432129

[b23] Zheng J. Energy metabolism of cancer: Glycolysis versus oxidative phosphorylation. Oncol. Lett. 4, 1151–1157 (2012). 23226794 10.3892/ol.2012.928 PMC3506713

[b24] Newsholme P. *et al.* Glutamine metabolism by lymphocytes, macrophages and neutrophils: Its importance in health and disease. J. Nutr. Biochem. 10, 316–324 (1999). 15539305 10.1016/s0955-2863(99)00022-4

[b25] Jha A. K. *et al.* Network integration of parallel metabolic and transcriptional data reveals metabolic modules that regulate macrophage polarization. Immunity 42, 419–430 (2015). 25786174 10.1016/j.immuni.2015.02.005

[b26] Green D. R., Galluzzi L. & Kroemer G. Mitochondria and the Autophage-inflammation-cell death axis in organismal aging. Science 333, 1109–1112 (2011). 21868666 10.1126/science.1201940 PMC3405151

[b27] Kaczor J. J. *et al.* The effect of aging on anaerobic and aerobic enzyme activities in human skeletal muscle. J. Gerontol. A. Biol. Sci. Med. Sci. 61, 339–344 (2006). 16611699 10.1093/gerona/61.4.339

[b28] Lee C.-K., Klopp R. G., Weindruch R. & Prolla T. A. Gene expression profile of aging and its retardation by caloric restriction. Science. 3, 1290–1293 (1994). 10.1126/science.285.5432.1390 10464095

[b29] Kohut M. L. *et al.* Age effects on macrophage function vary by tissue site, nature of stimulant, and exercise behavior. Exp. Gerontol. 39, 1347–1360 (2004). 15489058 10.1016/j.exger.2004.07.001

[b30] Bogle R. G., Baydoun A. R., Pearson J. D., Moncada S. & Mann G. E. L-Arginine transport is increased in macrophages generating nitric oxide. Biochem. J. 284, 15–18 (1992). 1599394 10.1042/bj2840015 PMC1132690

[b31] Kurko J. *et al.* Dysfunction in macrophage toll-like receptor signaling caused by an inborn error of cationic amino acid transport. Mol. Immunol. 67, 416–425 (2015). 26210182 10.1016/j.molimm.2015.07.006

[b32] Yeramian A. *et al.* Arginine transport via cationic amino acid transporter 2 plays a critical regulatory role in classical or alternative activation of macrophages. J. Immunol. 176, 5918–5924 (2006). 16670299 10.4049/jimmunol.176.10.5918

[b33] Ruiz-García A. *et al.* Cooperation of adenosine with macrophage Toll-4 receptor agonists leads to increased glycolytic flux through the enhanced expression of PFKFB3 gene. J. Biol. Chem. 286, 19247–19258 (2011). 21464136 10.1074/jbc.M110.190298 PMC3103303

[b34] Adanin S. *et al.* Inhibiting adenosine deaminase modulates the systemic inflammatory response syndrome in endotoxemia and sepsis. Am. J. Physiol. Regulatory Integrative Comp. Physiol 282, R1324–R1332 (2002). 10.1152/ajpregu.00373.2001 11959672

[b35] Hotamisligil G. S. & Erbay E. Nutrient sensing and inflammation in metabolic diseases. Nat. Rev. Immunol. 8, 923–934 (2008). 19029988 10.1038/nri2449 PMC2814543

[b36] Evans S. S., Repasky E. a. & Fisher, D. T. Fever and the thermal regulation of immunity: the immune system feels the heat. Nat. Rev. Immunol. 15, 335–349 (2015). 25976513 10.1038/nri3843 PMC4786079

[b37] Norman D. C. Fever in the elderly. Infect. Dis. Clin. North. Am. 10, 148–151 (1996). 10.1016/s0891-5520(05)70288-9 8698997

[b38] Lochmiller R. L. & Deerenberg C. Trade-offs in evolutionary immunology: just what is the cost of immunity? OIKOS 88, 87–98 (2000).

[b39] Brownie S. Why are elderly individuals at risk of nutritional deficiency? Int. J. Nurs. Pract. 12, 110–118 (2006). 16529597 10.1111/j.1440-172X.2006.00557.x

[b40] Hurson M., Regan M. C., Kirk S. J., Wasserkrug H. L. & Barbul A. Metabolic effects of arginine in healthy elderly population. JPEN J. Parenter. Enteral. Nutr. 19, 227–230 (1995). 8551652 10.1177/0148607195019003227

[b41] Davies J. Q. & Gordon S. Isolation and culture of murine macrophages. Methods Mol. Biol. 290, 91–103 (2005). 15361657 10.1385/1-59259-838-2:091

[b42] Weischenfeldt J. & Porse B. Bone marrow-derived macrophages (BMM): isolation and applications. CSH Protocol 3, 1–7 (2008). 10.1101/pdb.prot5080 21356739

[b43] Fei F., Bowdish D. M. E. & McCarry B. E. Comprehensive and simultaneous coverage of lipid and polar metabolites for endogenous cellular metabolomics using HILIC-TOF-MS. Anal. Bioanal. Chem. 406, 3723–3733 (2014). 24714971 10.1007/s00216-014-7797-5 PMC4026627

[b44] Trapnell C. *et al.* Differential gene and transcript expression analysis of RNA-seq experiments with TopHat and Cufflinks. Nat. Protoc. 7, 562–578 (2012). 22383036 10.1038/nprot.2012.016 PMC3334321

[b45] Smith C. A., Want E. J., O’Maille G., Abagyan R. & Siuzdak G. XCMS: processing mass spectrometry data for metabolite profiling using nonlinear peak alignment, matching, and identification. Anal. Chem. 78, 779–787 (2006). 16448051 10.1021/ac051437y

[b46] Kuhl C., Tautenhahn R. & Neumann S. LC-MS Peak Annotation and Identification with CAMERA. 1–14 (2010).

