# Supplementary material for: Age-associated metabolic dysregulation in bone marrow-derived macrophages stimulated with lipopolysaccharide
Source: Sci Rep. 2016 Mar 4;6:22637. doi: 10.1038/srep22637 (PMC4778050; doi:10.1038/srep22637)
Supplement: Supplementary Information [file srep22637-s1.pdf]

Age-associated metabolic dysregulation in bone marrow-derived macrophages stimulated with lipopolysaccharide

Fan Fei [1,2], *Keith M. Lee* [2], *Brian E. McCarry* [1], *Dawn M. E. Bowdish* [2*],

[1] Department of Chemistry and Chemical Biology, McMaster University, Hamilton, Canada L8S4M1

[2] Department of Pathology and Molecular Medicine, Michael G. DeGroote Institute for Infectious Disease Research, McMaster University, Hamilton, Canada L8N3Z5

*Corresponding author: Email: bowdish@mcmaster.ca; Phone: (+1)905-525-9140 ext22313; Fax: (+1) 905-522-6750

1.1 GC-MS

For the targeted GC-MS analysis, metabolic intermediates of glycolysis, the citric acid cycle (TCA), the γ-aminobutyric acid (GABA) shunt and the urea cycle were included except acetyl-CoA, succinyl-CoA, succinyl-semialdehyde, ornithine and arginine (Table S1). The former three metabolites were excluded from the list due to limitations of GC-MS or lack of authentic standards. Ornithine and arginine were measured using LC-MS instead of GC-MS because arginine is partially converted to ornithine during trimethylsilylation step with MSTFA and 1% TMCS[1] (Fig. S1). The percentage of conversion is not linearly proportional to the arginine concentration. Therefore, it is important to analyze arginine and ornithine with LC-MS instead of GC-MS technique for better and reliable data quality.

TMCS is a silylation catalyst that increases the reactivity of other silylation reagents. 1% TMCS is important for trimethylsilylation derivation for GC-MS analysis for analyte with functional groups of low reactivity such as hydroxyl and amide groups. The reactivity of trimethylsilylatioin between functional groups and MSTFA follows the alcohol>phenol>carboxyl>amine>amide/hydroxyl order with primary amine/alcohol being more reactive than tertiary. Multiple adducts with different number of trimethylsilyl ester derivatives can be observed in the study for one metabolite (e.g. GABA). Multiple peaks can also be noted for glucose from direct derivitization due to varying structural forms such as cyclic or open-chain structure[2].

All targeted metabolites were found to undergo continuous derivitization while queued on the autosampler at ambient temperature. Cooling the autosampler to below 5ºC quenched the ongoing derivitization for most targeted metabolites. Therefore, it is critical to analyze the macrophage extract by GC-MS immediately after derivitization. When not possible, the derivatized sample was stored in -20 or -80ºC until GC-MS analysis, for which no further derivation was noted.

Anthracenemethanol was selected as internal standard for GC-qMS study because it can be detected in derivatized and non-derivatized form. In addition to be used for peak area normalization, it is used to monitor the completeness of the derivation procedure by monitoring the ratio between m/z 208 and m/z 280.

Table S1 Target Metabolites for GC-qMS analysis

No.	Metabolites	Retention time (min)	Quantifying ion (m/z)	Qualifying ion (m/z)
**1**	lactate	5.39	147	73, 117, 219
**2**	alanine	5.68	116	73, 147
**3**	pyruvate	5.80	217	73, 147
**4**	γ-aminobutyric acid (GABA-diTMS)	9.36	102	73, 147, 232
**5**	proline	9.36	142	73, 216
**6**	fumarate	10.21	245	73, 147
**7**	succinate	10.53	147	73, 247
**8**	GABA-triTMS	13.63	174	73, 147, 304
**9**	malate	13.69	233	73, 147, 335
**10**	methionine-d_3-monoTMS	14.09	107	73, 224
**11**	aspartate	14.31	232	73, 100, 147
**12**	Oxaloacetate-1	15.38	333	73, 147
**13**	Oxaloacetate-2	15.72	333	73 147
**14**	ornithine-triTMS	15.46	142	70, 73, 348
**15**	glutamate	16.53	246	73, 128, 147, 156
**16**	α-ketoglutarate	16.78	347	73, 147, 318
**17**	phenylalanine-d_8	17.39	219	73, 147
**18**	ornithine-tetraTMS	19.06	142	73, 174, 420
**19**	glutamine	20.69	156	73, 147, 245
**20**	citrate	20.74	273	73, 147, 465
**21**	Arginine [a]	20.86	256	73, 147, 157
**22**	glucose-1	20.96	204	73, 147, 191, 217
**23**	glucose-2	22.42	204	73, 147, 191, 217
**24**	2-Phosphoglyceric acid (2-PG)	21.14	299	73, 147, 387, 459
**25**	isocitrate	21.16	273	73, 147, 245, 465
**26**	3-Phosphoglyceric acid (3-PG)	21.61	299	73, 357, 387, 459
**27**	citrulline-tetraTMS	24.36	188	73, 100, 346
**28**	fructose-6-phosphate (F6P)	28.30	315	73, 387, 589
**29**	tryptophan-d_5	29.70	207	73, 291
**30**	glucose-1-phosphate (G1P)	30.94	387	73, 204
**31**	Glucose-6-phosphate	31.14	387	73, 315, 589
**32**	anthracenemethanol	31.38	280	
**33**	argininosuccinate	35.94	149	73, 563, 578

Table S2 Single-ion-monitoring (SIM) method for GC-qMS

SIM region	Time (min)	Ions (m/z)
**1**	5.0-7.0	73, 116, 117, 147,217, 219
**2**	7.0-10.0	73, 102, 142, 147, 216, 232
**3**	10.0-11.5	73, 147, 245, 247
**4**	11.5-14.9	73, 100, 107, 147, 174, 224, 232, 233, 304, 335
**5**	14.9-16.0	70, 73, 142, 147, 157, 173, 179, 218, 296, 333, 348
**6**	16.0-18.0	73, 128, 147, 156, 200, 219, 246, 318, 347
**7**	18.0-20.0	73, 142, 174, 420
**8**	20.0-23.5	73, 147, 156, 157, 191, 204, 217, 245, 256, 273, 299, 357, 387, 459, 465
**9**	23.5-26.0	70, 73, 100, 188, 346
**10**	26.0-30.5	70, 73, 100, 204, 207, 291, 315, 387, 589
**11**	30.5-33.0	73, 204, 208, 280, 387
**12**	33.0-40.0	73, 149, 563, 578

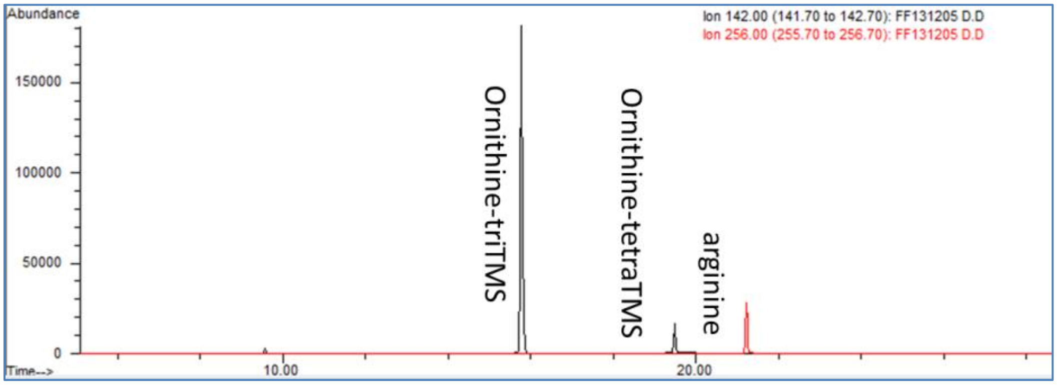

Fig. S1 The GC-EI-MS chromatogram of arginine standard

1.2 Data quality control

The final comprehensive murine macrophage metabolic profile data set acquired by LC-MS consisted of 2125 metabolite features, of which 121 metabolites were identified. The extraction efficiency was 76-83% based on L-methinione-d_3, a standard for recovery determination (RS). The biological variance was 20-35% and 29-40% for macrophages derived from young or old mice, respectively (Table S3). The biological variance was consistently greater for macrophages derived from old mice for all treatment conditions. Post column addition using gly-phe had shown that there was no ion suppression noted in both ESI+ and ESI- modes except between 0.6-1.3 min and 7.1-7.8 min of the chromatogram. Features eluted between 0.6-1.3 were removed during data reduction process. Features eluted between 7.1-7.8 were normalized to L-phenylalanine-d8, which also eluted in the same region, to correct for ion suppression. The comprehensive data set was visualized by principle component analysis (PCA) to assess the LC-MS instrumentation reproducibility. The pooled samples were tightly clustered in the centre of the score plot, which indicating the technical variability was minimal in comparison to the

biological variance and the pooled sample resembled the average of all samples from various treatment conditions. Of the comprehensive data set, 69.9% of the features were normally distributed; the metabolites included in the targeted data set were all normally distributed.

Table S3 Coefficient of variance of macrophage metabolites from young or old mice.

		macrophages	
Treatment groups	Treatments	Young mice	Old mice
Group 1	4 hr LPS	35%	40%
Group 2	16 hr LPS	35%	40%
Group 3	Tolerance	23%	43%
Group 4	Recovery	20%	29%
Group 5	Control 1	30%	40%
Group 6	Control 2	32%	43%

Table S4 Summary of total number of metabolite features extracted from LC-MS data after XCMS, CAMERA and data reduction

Total No. features	ESI +	ESI -
XCMS	2060	2224
Total	4284	
k' < 0.7 [a]	198	125
IS/RS	5	5
Salts (sodium formate)	14	14
Percentage variance >30% in pooled samples	596	
**Finalized feature list**	**2125**	

[a] Retention factor $k' = \frac{t_R - t_0}{t_0}$, t_R is the retention time of metabolic feature and t_0 is the time required for mobile phase to pass through the column

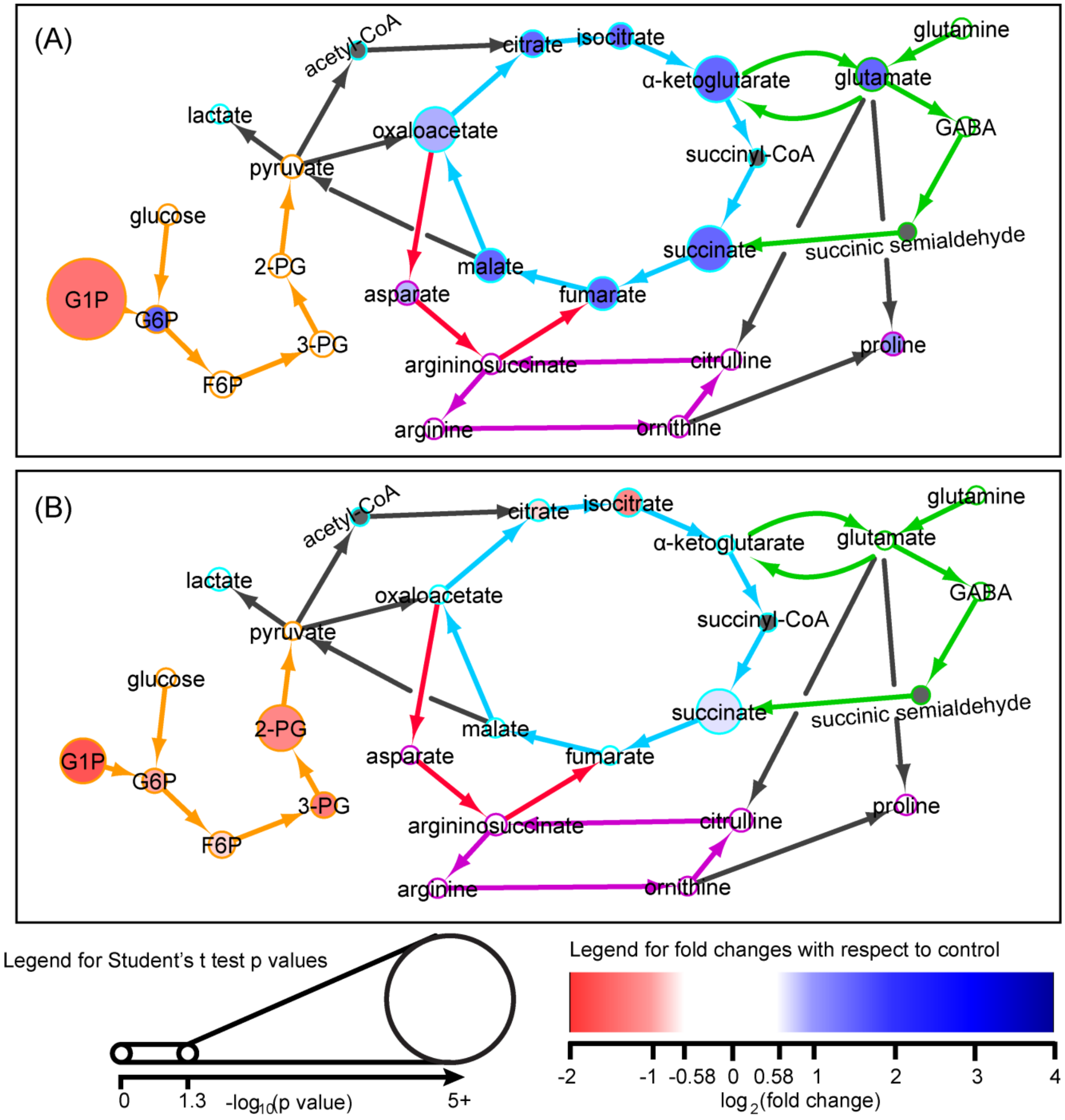

Fig. S2 The enrichment map of glycolysis, the TCA cycle, the GABA shunt, and the urea cycle intermediates during 4 hrs of LPS stimulation in bone marrow-derived macrophages from (A) young mice or (B) old mice as compared to corresponding unstimulated macrophages. The network of metabolite interactions was built based on the BioCyc database and pathway published from Jha et al.[3]. The node size is proportional to the significance of metabolite changes compared to the control. The colors of the nodes indicate the $\log_2$(fold changes) of metabolite levels of each experimental condition compared to the control with a decrease colored in red and an increase colored in blue. Acetyl-CoA, succinyl-CoA and succinic semialdehyde are not detected and therefore are labelled in grey. The glycolysis pathway is labelled in orange; the TCA cycle is labelled in blue; the GABA shunt is labelled in green; the glutamate-argininosuccinate shunt is labelled in red; the urea cycle is labelled in purple.

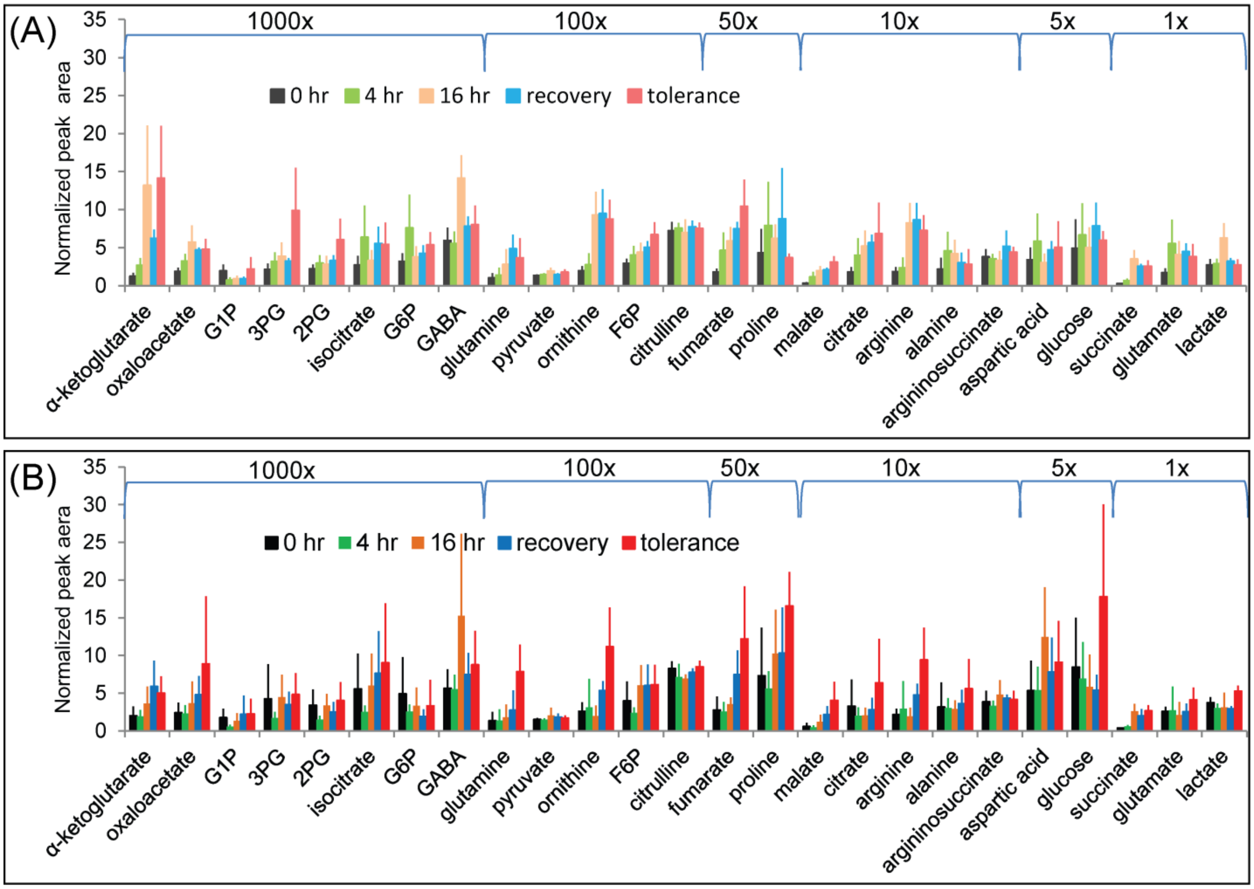

Fig. S3 The relative abundances of the 25 targeted intracellular metabolites from macrophages of (A) young and (B) old mice from five different experimental conditions as acquired by GC-MS. The samples were performed in sextuplicate with three biological replicates and two culture replicates. The error bar represents one standard deviation. The relative abundances of some metabolites were amplified by 5, 10, 50, 100, or 1000 fold to scale to the figure. G1P, glucose-1-phosphate; 3PG, 3-phosphoglycerate; 2PG, 2-phosphoglycerate; G6P, glucose-6-phosphate; GABA, γ-aminobutyric acid

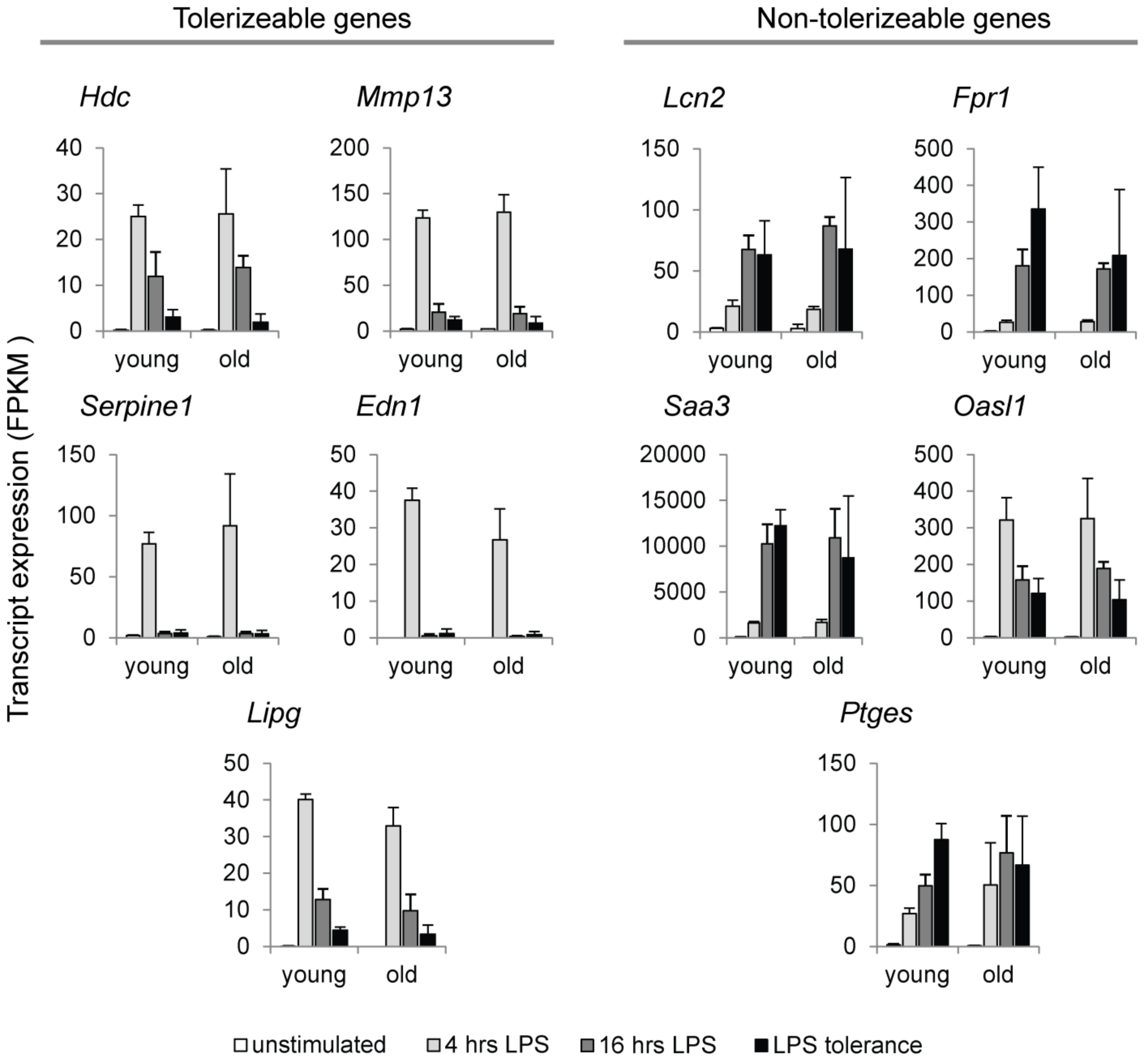

Fig. S4 The gene expression of tolerance and non-tolerance associated genes identified by Foster et al.[4]. The expression of these genes were acquired from unstimulated (0 hr), 4 hrs and 16 hrs of LPS stimulation, and LPS tolerant macrophages from either young or old mice.

References

1. Halket, J. M. *et al.* Chemical derivatization and mass spectral libraries in metabolic profiling by GC/MS and LC/MS/MS. *Journal of experimental botany* **56**, 219–43 (2005).

2. Fiehn, O., Kopka, J., Trethewey, R. N. & Willmitzer, L. Identification of uncommon plant metabolites based on calculation of elemental compositions using Gas chromatography and quadrupole mass spectrometry. *Anal. Chem.* **72**, 3573–3580 (2000).

3. Jha, A. K. *et al.* Network integration of parallel metabolic and transcriptional data reveals metabolic modules that regulate macrophage polarization. *Immunity* **42**, 419–430 (2015).

4. Foster, S. L., Hargreaves, D. C. & Medzhitov, R. Gene-specific control of inflammation by TLR-induced chromatin modifications. *Nature* **447**, 972–978 (2007).
